# Model Milk

**Published:** 1920-10-02

**Authors:** 


THE PUBLIC WELL-BEING—(*continued*).

### Model Milk.

This week the price of milk has risen again. It would be good if this could be attributed to the added expense of producing a purer article, but, unfortunately, that is not the explanation. The quality of milk is receiving a great deal of attention nowadays, but it is a slow process to bring much-needed reforms into practice even in so important a thing as milk. There are some, however, who are showing the way, and of these Mr. Wilfred Buckley has not only preached reform, but has practised it. Two descriptions of his methods have recently appeared—one in connection with the supply of milk to the Yarrow Convalescent Home for the children of middle-class parents, and the other in connection with a farm near Basingstoke. It seems so near an approach to perfection that it is well that this model method should be widely known and appreciated. A medical officer of health, writing in *Maternity and Child Welfare*, describing the work at Moundsmere Farm, Basingstoke, says the cows before milking are groomed; the hair on the udder is kept short with clippers, the brush of the tail cut to knee-length. The milkers wash their hands before starting milking; they wear overalls and caps and are careful not to press their heads against the cow's side; in this way they avoid disturbing any dust or loose hair. The first two squirts from each teat contain a large number of germs; this fore-milk is discarded. If the milker's hand is wet there is a danger of a drop of fluid containing germs falling into the milk, so at Moundsmere he milks with a dry hand. The milk stools are kept clean and, most important of all, every utensil employed is sterilised before use. The buckets used for milking have a covered seamless top; the hole is placed at the side so as to prevent any hair or dust falling into the milk. To prevent dust and dirt being carried into the bottling-room by the milkers, the milk is poured into a small sterilised receiving tank fixed to the outside of the wall; from this it passes by a tube through the wall to a filter placed over a tank, out of which the milk flows over the cooler, a framework of tubes through which cold water circulates. The milk falls from the cooler into a bottle; when a dozen or so bottles have been filled the dairymaid places a sterilised disc in the neck, by means of a small automatic hand-machine; then she places a paper cap over the top of the bottle and, with another hand-machine fitted with wire, fastens the cap round the neck with wire. The bottles are placed in a partitioned box with half a shovelful of ice which has been lying on the floor of the dairy, keeping the room cool. The dairymaid wears a clean overall, a handkerchief covers her hair; her hands and nails are kept scrupulously clean.

The bottles are washed by a machine, first with caustic alkali, which is rinsed out with water in a bath, then revolving brushes clean the inside and outside; after this the bottles are inverted over a jet of clean water for a final rinsing before sterilisation.

The average sample of London milk contains 3,500,000 germs in a cubic centimetre, Moundsmere milk a number varying from 5,200 to none at all, the average of twenty-three samples being 1,100.

The price of this milk is 7d. a pint in Manchester, 7½d. a pint, or 1s. 1d. a quart, in London. The difference between the price of this and the ordinary milk is not much considering the difference in the conditions of production, and we can speak with knowledge of the rich quality.

The system at the dairy attached to Yarrow Convalescent Home is similar, and produces about the same results in the reduction of bacteria per cubic centimetre. Mr. Buckley's advice was taken by Dr. Moon, the medical officer of the Home, in setting up this dairy for the children.

